# The 20-million-year old lair of an ambush-predatory worm preserved in northeast Taiwan

**DOI:** 10.1038/s41598-020-79311-0

**Published:** 2021-01-21

**Authors:** Yu-Yen Pan, Masakazu Nara, Ludvig Löwemark, Olmo Miguez-Salas, Björn Gunnarson, Yoshiyuki Iizuka, Tzu-Tung Chen, Shahin E. Dashtgard

**Affiliations:** 1 Department of Geosciences, National Taiwan University, P.O. Box 13-318, Taipei, 106 Taiwan; 2 Department of Biological Sciences, Faculty of Science and Technology, Kochi University, Kochi, 780-8520 Japan; 3 Department of Stratigraphy and Palaeontology, University of Granada, 18002 Granada, Spain; 4 Department of Physical Geography and Quaternary Geology, Stockholm University, 106 91, Stockholm, Sweden; 5 Institute of Earth Sciences, Academia Sinica, Taipei, 11529 Taiwan; 6 Department of Earth Sciences, University of Gothenburg, Box 460, 405 30 Göteborg, Sweden; 7 Department of Earth Sciences, Simon Fraser University, Burnaby, BC V5A 1S6 Canada

**Keywords:** Evolution, Ecology, Environmental sciences

## Abstract

The feeding behavior of the giant ambush-predator “Bobbit worm” (*Eunice aphroditois*) is spectacular. They hide in their burrows until they explode upwards grabbing unsuspecting prey with a snap of their powerful jaws. The still living prey are then pulled into the sediment for consumption. Although predatory polychaetes have existed since the early Paleozoic, their bodies comprise mainly soft tissue, resulting in a very incomplete fossil record, and virtually nothing is known about their burrows and behavior beneath the seafloor. Here we use morphological, sedimentological, and geochemical data from Miocene strata in northeast Taiwan to erect a new ichnogenus, *Pennichnus*. This trace fossil consists of an up to 2 m long, 2–3 cm in diameter, L-shaped burrow with distinct feather-like structures around the upper shaft. A comparison of *Pennichnus* to biological analogs strongly suggests that this new ichnogenus is associated with ambush-predatory worms that lived about 20 million years ago.

## Introduction

Eunicid polychaetes (Family Eunicidae) are well-known for their long, segmented bodies, and strong denticulated maxillae^1^. They are widely distributed in tropical to temperate shallow marine to intertidal environments in the Atlantic, Indian, and Pacific Oceans^1–4^. One of the largest eunicids, the so called “Bobbit worm” (*Eunice aphroditois*), has fascinated the public since its appearance in November 2017 in British Broadcasting Corporation’s “Blue Planet II”. However, because burrowing Bobbit worms only extend a minor portion of their body outside of the sediment, observations on their behavior beneath the seafloor are difficult. As a result, previous studies mainly focused on anatomy and ethology of this and other eunicids^1,3,5^, and little is known of their burrow morphology.

Bobbit worms can grow up to 3 m long and 2 cm in diameter^3,5^. They are ambush predators that feed on fish, bivalves, and other annelids^6,7^. Upon reaching sexual maturity, some Bobbit worms construct a permanent mucus-lined burrow in the seafloor^8^, and some use an antenna to simulate a wormlike motion for attracting prey^7^. When potential prey comes within striking distance, the Bobbit worm rapidly thrusts out of its burrow and grabs the prey with its eversible pharynx and sharp maxillae^6^. The still living animal is then dragged down into the burrow for consumption, and the desperate attempts of the prey to escape commonly leads to disturbance and collapse of the sediment around the burrow opening^7^.

Polychaetes emerged early in the Cambrian, and jaws of possible raptorial worms have been described from early Paleozoic strata^9,10^. Consequently, burrows of raptorial polychaetes could potentially occur in strata as old as the early Paleozoic. Most of the existing trace fossils produced by polychaetes, such as *Planolites*, *Rosselia*, *Cylindrichnus*, and *Arenicolites*, represent deposit-feeding, detritus-feeding, or filter-feeding behaviors, rather than predatory ones^11–14^. Trace fossils associated with predatory behavior, such as *Piscichnus*, are primarily produced by vertebrates including fish, rays, and marine mammals that extract prey by jetting water into the sediment^15–18^. Only a few trace fossils are described as preserving hunting behaviors of invertebrates (e.g., trilobites, gastropods)^19,20^, and trace fossils produced by subsurface-dwelling ambush predators have not been identified previously. Herein, we present morphological, sedimentological and geochemical data of giant L-shaped burrows in the Miocene Taliao Formation of northeast Taiwan. These data are compared to modern marine environments of the northwest Pacific and to biological analogs, and an argument is presented to suggest that the L-shaped burrows record the hunting behaviors of Miocene Bobbit worms.

**Figure 1:**
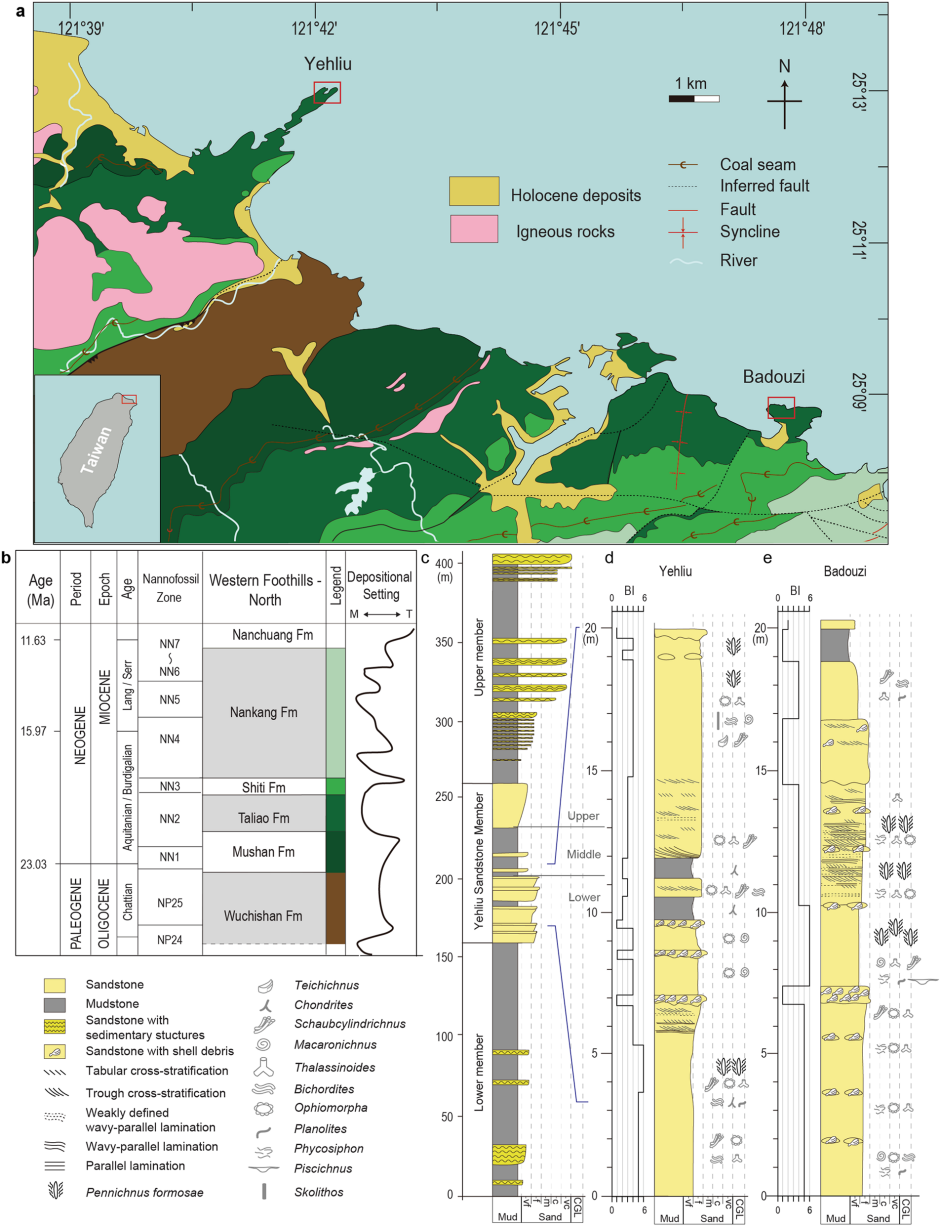
Geological setting and study area in northern Taiwan. (**a**) Geological map of northern Taiwan with measured section locations (red rectangles). The map is modified from Taipei and Shuanghsi geological maps published by Central Geological Survey, MOEA^26,27^. Polygon map colors correspond to the legend in (**b**). (**b**) The chronostratigraphic chart of Oligocene to Miocene strata in northern Taiwan. The depositional settings curve distinguishes terrestrial (T)-from marine (M)-dominated strata^15^. Lang/Serr is the abbreviations of Langhian/Serravallian; Fm is the abbreviation of Formation. (**c**) Schematic stratigraphic section of the Taliao Formation. Detailed outcrop logs of the studied strata are shown for (**d**) Yehliu and (**e**) Badouzi with the associated legend included in the bottom left. Interpreted Bobbit worm burrows, *Pennichnus formosae*, are concentrated in certain layers (more symbols = higher abundance).

The island of Taiwan uplifted during the Penglai Orogeny, which recorded the collision between the Luzon Arc on the Philippine Sea Plate and Eurasian Plate starting about 5 million years ago^21,22^. Along the Northeast Coast of Taiwan, late Oligocene to Pliocene passive margin strata consist of shallow marine to coastal swamp deposits, and record a number of transgressive–regressive cycles^23–25^; these deposits are exquisitely exposed due to a series of imbricated thrust faults (Fig. 1a,b).

The Yehliu Sandstone Member is part of the early Miocene Taliao Formation (~ 22–20 Ma) and is extensively exposed along the Northeast Coast^26,27^. It is interpreted as recording deposition in lower shoreface to upper offshore paleoenvironments^23,28–30^, and is encased between mudstone-dominated members of the Taliao Formation^29^ (Fig. 1c). The lower part of the Yehliu Sandstone Member comprises highly bioturbated sandstone with numerous marine trace fossils, and with intermittent scoured sandstone beds containing shell debris (Fig. 1c–e; Supplementary Fig. S1). Within the bioturbated sandstone layers at both Yehliu Geopark and Badouzi promontory (Fig. 1c), large, L-shaped burrows, named herein as *Pennichnus formosae* n. isp. (the complete systematic ichnology can be found as Supplementary Discussion 1) are locally abundant.

## Results

### Preservation and occurrence of *Pennichnus*

At Yehliu Geopark (Fig. 1d), *Pennichnus* are preserved in very fine- to fine-grained sandstone. They co-occur with abundant *Bichordites*, *Ophiomorpha*, *Phycosiphon*, *Planolites*, and *Schaubcylindrichnus*, and uncommon *Macaronichnus, Teichichnus*, and *Skolithos*. This trace-fossil assemblage is best described as a proximal *Cruziana* Ichnofacies^31,32^ and indicates deposition in a lower shoreface to offshore paleoenvironment. At Badouzi promontory, *Pennichnus* are also concentrated in very fine- to fine-grained sandstone (Fig. 1e), but co-occur with *Thalassinoides*, *Phycosiphon*, *Macaronichnus*, *Schaubcylindrichnus*, *Planolites*, *Ophiomorpha* and *Piscichnus*. The trace-fossil assemblage at Badouzi correlates to the *Cruziana* Ichnofacies, and suggests deposition occurred in an offshore paleoenvironment^31,32^.

The sandstone beds containing *Pennichnus* at both Yehliu and Badouzi are at the same stratigraphic position (Fig. 1c–e); however, Yehliu is considered to preserve deposition of a higher-energy, shallower-water environment that reflects a northwestward shallowing paleo-bathymetry^29^. When *Pennichnus* co-occurs with *Phycosiphon*, *Schaubcylindrichnus*, and *Thalassinoides*, the number of specimens reaches 1.7 m^−2^ (between 7.5 and 9.9 m, Fig. 1e); this is the highest density among all *Pennichnus*-bearing strata (Supplementary Fig. S2). When *Pennichnus* co-occurs with *Ophiomorpha*, *Teichichnus*, and *Skolithos*, the number of specimens drops to 0.5 m^−2^ (17.8–19.5 m, Fig. 1d). Two other zones with abundant *Pennichnus* (3.7–5.2 m, Fig. 1d and 11–13.5 m, Fig. 1e) exhibit densities of 0.7 m^−2^ and 0.8 m^−2^, respectively (Supplementary Fig. S2).

### Morphology of *Pennichnus*

The morphology of *Pennichnus* is reconstructed using 319 specimens observed in Yehliu Geopark and Badouzi promontory. The overall configuration of *Pennichnus* is that of an L-shaped, smooth-walled burrow that can exceed 2 m in length. In vertical section, the burrow diameter is typically 2.5 ± 0.6 cm at the top of the burrow, and this tapers down-burrow to 2.0 ± 0.6 cm near the end of the horizontal section (Supplementary Fig. S3). Most examples of *Pennichnus* are filled with sediment similar to the host sediment or with sandstone that is less muddy than the host sediment, and this suggests that the burrows were passively filled after the trace maker either abandoned the burrow or died^33^.

**Figure 2:**
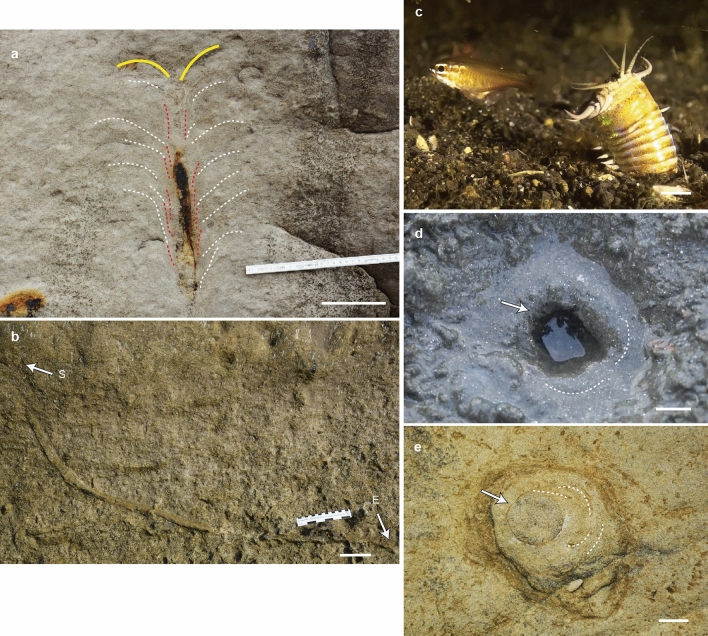
Photos of *Pennichnus*, *Eunice aphroditois* and its burrow. (**a**) Vertical section of the upper part of *Pennichnus formosae*. The funnel top (yellow line), disturbed zone (dashed red lines) and feather-like collapse structures (dashed white lines) are visible. (**b**) Vertical section of the lower part of *Pennichnus formosae* (S = start and E = end of the burrow exposure). (**c**) Photo of *Eunice aphroditois* (Bobbit worm) (photo courtesy of Ms. Chutinun Mora). (**d**) *Eunice aphroditois* burrow entrance in the intertidal zone (photo courtesy of Ms. Chutinun Mora). (**e**) Plan view of the upper part of *Pennichnus formosae.* In both (**d**) and (**e**), the shaft of the burrow (white arrow) and surrounding concentric layers (dashed white lines) are visible. Scale bar: (**a–b**) = 10 cm; (**c–e**) = 1 cm. Photos without drawings are included in Supplementary Fig. S5.

*Pennichnus* consists of three distinct parts (Supplementary Fig. S4). The upper part is composed of a vertical shaft with a funnel aperture surrounded by inverted, cone-in-cone structures, and which show a feather-like appearance in vertical cross-section (referred to as feather-like structures; Fig. 2a; Supplementary Figs. S5 and S6). The vertical part of the burrow comprises about 40% of the total burrow length, and is connected to a gently curving middle section (10% of the total length), and then a lower horizontal section (50% of the total length; Fig. 2b; Supplementary Fig. S5). Downward along the burrow, the feather-like structures gradually disappear (Fig. 2a; Supplementary Fig. S5). In bedding plane view, the funnel aperture displays a circular to oval shape surrounded by concentric laminations, and this is similar to the opening around modern Bobbit worm burrows (Fig. 2c–e; Supplementary Fig. S5).

Among the 157 well-exposed *Pennichnus* specimens, 82.2% of specimens show no aberrant morphological features, while 17.8% show one or two (Supplementary Fig. S7; Supplementary Table 1). Specifically, 12.1% of well exposed *Pennichnus* specimens are preserved with a homogenous burrow fill penetrated by an inner tube, 3.2% display evidence of active backfilling in parts of the burrow but without an inner tube, and 2.5% of them display both evidence of active backfilling, and an inner tube. (Supplementary Table 1). The fill of the inner tube is homogenous. Similar thin tubes were observed in the sediment surrounding *Pennichnus* with no apparent connection to the burrows and this suggest that when found inside the trace fossil, they may represent secondary occupation of an abandoned burrow rather than comprising part of *Pennichnus*.

### Geochemical characterization of *Pennichnus*

**Figure 3:**
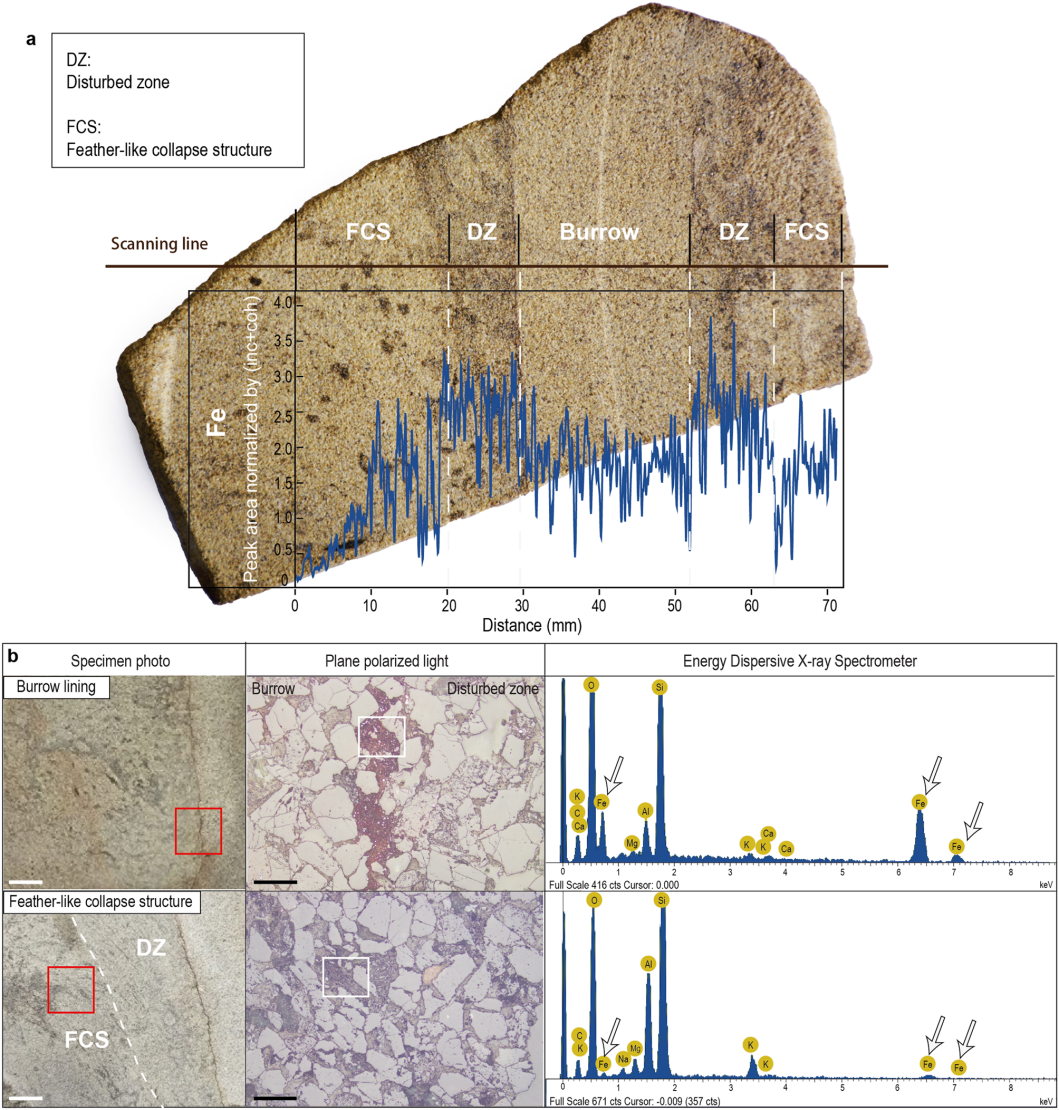
Iron (Fe) variation across the *Pennichnus* specimen. (**a**) Variations in Fe across *Pennichnus formosae* measured by an Itrax Multiscanner. Fe increases at the burrow lining and remains high through the surrounding disturbed zone (DZ). (**b**) SEM–EDS analyses of thin sections of the burrow lining and feather-like collapse structures (FCS). The red rectangles mark the thin section locations and the white rectangles mark the part of the thin section analyzed by EDS. The white arrows show that the burrow lining is enriched in Fe but the feather-like collapse structures are not. White scale bar = 1 cm; black scale bar = 200 µm.

X-ray fluorescence (XRF) core scanning reveals that there is an abrupt increase in iron (Fe) in the burrow lining and the deformed sediment surrounding the burrow (Fig. 3a). The deformed sediment corresponds to the innermost part of the feather-like structures (Fig. 2a). Energy Dispersive X-ray Spectrometer (EDS) analysis done using scanning electron microscope also shows low levels of Fe in the outer feather-like structures and higher Fe in the innermost part (Fig. 3b). Thus, the feather-like structures are divided into: (1) an inner disturbed zone, which is the deformed sediment immediately surrounding the burrow and with a high Fe concentration; and (2) an outer feather-like collapse structure zone with deformed sediment and low Fe concentration.

## Discussion

Iron variation laterally through the upper shaft (Fig. 3) suggests that *Pennichnus* were produced by mucus-secreting animals which repeatedly rebuilt the burrow opening through time. When marine invertebrates, especially soft-bodied infauna, construct their burrows, they usually secrete mucus to strengthen the burrow wall. Organic-rich compounds in the mucus then attract microbes such as sulfate-reducing bacteria that feed on the carbon leading to the formation of reducing conditions which triggers iron sulfide to precipitate in the lining^34–37^. During uplift and erosion, the diagenetically altered burrows are exposed to oxidizing pore waters, and the iron sulfide is oxidized to iron hydroxides such as hematite, limonite or goethite^37^; this results in elevated Fe levels in the burrow lining and disturbed zone . In contrast, low Fe concentration in the feather-like collapse structures (Fig. 3) suggests that these features were not affected by mucus, but rather formed through the collapse of sediment surrounding the burrow opening.

There are multiple trace makers that can produce large, lined burrows like *Pennichnus* and the three most probable animal groups are infaunal shrimp; fast-burrowing, siphonated bivalves; and, large polychaetes. Shrimp are well known for constructing open burrow networks^38,39^ and sometimes their burrows contain an inner tube potentially constructed by juvenile shrimp that re-occupy abandoned or infilled burrows^40^. However, shrimp burrow networks constructed in water-saturated sand typically comprise complicated shapes, like mazes or boxworks, and have turn-around chambers^41,42^. As well, shrimp burrow walls in water-saturated sand are normally reinforced with mud pellets^38^. Although previous studies showed that sediment collapse near the opening of abandoned shrimp burrows can form nested funnels which look similar to the feather-like structures of *Pennichnus*^38,43^, the nested funnels in shrimp burrows only occur above the aperture, rather than along the margin of the upper burrow (Supplementary Fig. S8). As well, *Pennichnus* specimens lack pelletoidal wall reinforcement, branching structures or turn-around chambers, and this suggests that shrimp are probably not the producers of *Pennichnus*.

With the presence of a partial meniscate backfill and inner tube in some *Pennichnus* specimens, another possible trace maker are fast-burrowing siphon-feeding bivalves such as *Ensis directus* (Atlantic razor clam) and *Tagelus plebeius* (Stout razor clam)^44–47^.These bivalves can build burrows up to 70 cm long^46^, and potentially produce collapse structures surrounding the actively backfilled burrows^44,45,48^. The maximum burrowing depth of these organisms is controlled by the strength of the substrate, which increases with depth^44,45^. The increase in sediment strength could explain the horizontal orientation of the burrows at a certain depth. However, the circular cross-section of *Pennichnus* is at odds with the shape of fast-burrowing bivalves, which tend to generate more almond- or keyhole-shaped burrows^49^. Moreover, rapid-burrowing bivalves excavate the sediment through local fluidization around their bodies^44^, and this commonly results in sediment collapse in their wake^49^. When sediment collapses into the cavity left by the bivalves, it blurs the burrow wall, which produces a burrow morphology that is very distinctive from the clear burrow wall preserved in *Pennichnus*. Furthermore, only few *Pennichnus* specimens (17.8%) display a backfill and/or inner tube, and no bivalve shells were observed within any *Pennichnus* even though shell material is common in the surrounding sediment. This suggests that fast-burrowing bivalves are not the trace maker.

The morphology of *Pennichnus* suggests that the trace maker was a large, slender organism that supported the burrow with its body, similar to how polychaetes do^47^. In addition, feather-like collapse structures and a disturbed zone surrounding the burrow lining indicates extensive sediment disturbance at shallow depths. These morphological features of *Pennichnus* are consistent with the activities of an ambush predator, and hence, we hypothesize that giant polychaetes, such as Bobbit worms, are the most probable trace makers (Fig. 2c). Bobbit worms are ambush-predators, and require long burrows to accommodate their 2 to 3 m-long and 2 to 2.5 cm-diameter bodies^3^. Their burrow morphology (Fig. 2d) and hunting behavior in modern environments explains the funnel opening and feather-like structures observed in *Pennichnus*. After each feed, the ambush-predatory polychaete re-establishes its burrow opening resulting in an accumulation of mucus linings, and this explains the occurrence of a Fe-enriched, disturbed sediment zone around the upper shaft. Sediment disruption after the Bobbit worm’s retraction is also consistent with the feather-like collapse structures. The body movements of Bobbit worms, as with all polychaetes, are achieved by controlling hydrostatic skeletons and parapodia, which give them various ways to move, including crawling, undulation and peristalsis^50,51^. Sedentary burrowing polychaetes, including ambush predators, mostly employ peristaltic locomotion when they burrow^52^, which produces a smooth-walled burrow without sinuosity. Since polychaetes respire through their body wall^53^, they might burrow horizontally at depth to avoid low-oxygen pore waters. This may explain the horizontal development of *Pennichnus* in the lower part of the burrow. Alternatively, the increasing energy required to excavate sediment at depth^44,45^ may also prompt the trace maker to burrow horizontally in less compacted sediment.

**Figure 4:**
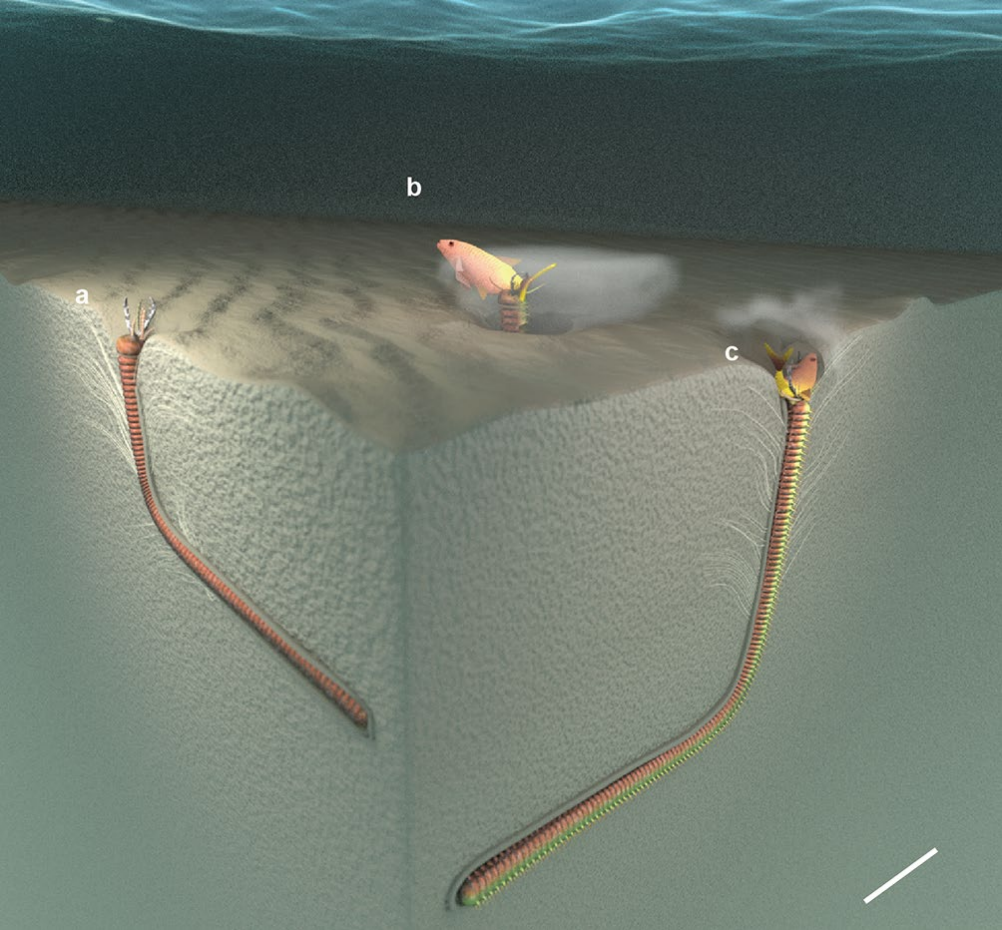
Schematic three-dimensional model of the feeding behavior of Bobbit worms and the proposed formation of *Pennichnus formosae*. (**a**) Bobbit worm sits inside the L-shaped burrow waiting for prey and (**b**) uses its strong jaws to catch the prey (e.g., fish) passing by the burrow opening (see video at https://www.mmoraa.com/video). (**c**) As the struggling prey is pulled into the burrow, the sediment collapses around the aperture to form feather-like collapse structures surrounding the upper burrow. Between the burrow and feather-like collapse structures is a disturbed zone caused by the repeated feeding action of the worm and burrow re-establishment that results in an accumulation of mucus lining over time. Scale bar = 30 cm.

To summarize, we hypothesize that about 20 million years ago, at the southeastern border of the Eurasian continent, ancient Bobbit worms colonized the seafloor waiting in ambush for a passing meal (Fig. 4). When prey came close to a worm, it exploded out from its burrow, grabbing and dragging the prey down into the sediment. Beneath the seafloor, the desperate prey floundered to escape, leading to further disturbance of the sediment around the burrow opening. The retreat of the ancient Bobbit worm and prey into the sediment caused the sediment to form the distinct feather-like collapse structures preserved in *Pennichnus formosae*. Upon consumption of its prey, the worm re-established its burrow, leading to the Fe-enriched disturbed zone surrounding the burrow wall.

In terms of the temporal and spatial distribution of *Pennichnus* trace makers, the eunicid polychaetes went through an evolutionary radiation in the Ordovician^54,55^ and giant jaw-bearing polychaetes have been described as far back as the Devonian^9^. In consequence, the record of *Pennichnus*, representing the predatory behavior of giant worms, may extend back to the Paleozoic. *Pennichnus* specimens described from Yehliu and Badouzi sections occur with trace fossil assemblages typical of the *Cruziana* Ichnofacies in a depositional environment close to storm-wave base level on a broad continental shelf. Thus, a similar offshore to shelf setting is probably where *Pennichnus* are likely to be found*.* However, we note that *Pennichnus* has not been described previously from strata that are interpreted to represent similar paleoenvironments as the Taliao Formation, suggesting that the occurrence of *Pennichnus* is controlled by a number of coinciding environmental conditions. Furthermore, although eunicid polychaetes are identified as the most probable trace maker in this study, we can’t exclude the possibility that other vermiform invertebrates besides eunicid polychaetes could produce *Pennichnus* when engaging in similar behavior.

In conclusion, the distinctive configuration, geometry and internal structure of the burrows described herein supports the erection of a new ichnogenus, *Pennichnus*, which records the behavior of giant predatory worms beneath the seafloor (Supplementary Discussion 1). The interpreted activities of the *Pennichnus* trace maker records a life and death struggle between predator and prey, and indirectly preserves evidence of more diverse and robust paleo-ecosystem than can be interpreted from the fossil and trace fossil record alone.

## Methods

### Morphological descriptions

The morphology of *Pennichnus* is reconstructed based on observations of 319 specimens in Yehliu Geopark and Badouzi promontory (Supplementary Fig. S1a,b). Each *Pennichnus* specimen was photographed and morphological parameters including vertical extent, horizontal extent, burrow diameter, and width of the feather-like structures were measured. Owing to its large size, nearly all specimens were only partially exposed, so we classified the exposed section as representing the upper (U), middle (M) and/or lower (L) part of the burrow (Supplementary Fig. S4). Measurements taken from the specimens preserving one of the three parts of the burrow were then compiled to quantify burrow morphology.

### Serial grinding

To overcome limited exposure and to better understand the internal structure of the burrow, selected specimens were ground down in the field using a portable sander (Makita DBO180 18v Random Orbit Sander). Specimens were ground layer-by-layer in increments < 1 mm providing serial sections through the trace fossil, and each successive layer was photographed (Supplementary Fig. S9).

### Computed tomography (CT) scanning

Five specimens were cut out of the outcrops for detailed analysis. To assess the 3D geometry of the burrow and surrounding feather-like structures, one specimen was examined by CT scanning (LightSpeed Ultra16; GE Healthcare Japan Corporation, Japan, 120 kV, 100 mA, slice thickness: 0.625 mm) at the Center for Advanced Marine Core Research (CMCR), at Kochi University in Japan. The CT scanning data were then processed using OsiriX imaging software (Supplementary Fig. S6c,d).

### SEM–EDS analysis

The mineral composition and clast arrangement of the burrow wall, fill, and host rock was assessed using petrographic thin sections studied with an optical microscope. However, the feather-like structures and wall-lining were difficult to identify under the microscope and were therefore analyzed in thin sections using a thermal field emission scanning electron microscope (JEOL FE-SEM: JSM-7100F) in the EPMA lab at Institute of Earth Sciences, Academia Sinica, Taipei, Taiwan. Chemical identification was carried out using an Energy Dispersive X-ray Spectrometer (EDS: Oxford Instruments X-max80 with INCA-350), equipped on the FE-SEM. Each sample was mounted, polished, and analyzed with FE-SEM and EDS under low vacuum conditions (50 Pa), using an acceleration voltage of 15 kV and beam current of 0.12 nA. The duration of EDS counting time was 15 s for each spot.

### X-ray fluorescence (XRF) analysis

In order to measure elemental variations between different parts of *Pennichnus*, two samples were studied with both an Itrax XRF Multiscanner (Department of Physical Geography, Stockholm University), and an Itrax Core Scanner (Department of Geoscience, National Taiwan University). Both machines are manufactured by Cox Analytical Systems.

### Construction of the stratigraphic columns

Stratigraphic columns including lithology, sedimentology, ichnology and body fossils were generated for the Yehliu Sandstone Member at both Yehliu and Badouzi. For each bed, grain size, sedimentary structures and bed thicknesses were recorded. Ichnological analysis focused on identifying trace fossils and assessing the degree of bioturbation with the bioturbation index^56^. The density of *Pennichnus* specimens was quantified by dividing the number of specimens by outcrop area.

### Nomenclatural acts

Following the rules of International Code of Zoological Nomenclature (ICZN)^57^, this study and its nomenclatural act are registered in ZooBank, the online registration system governed by ICZN, to validate the new ichnotaxon. By keying LSID (Life Sciences Identifier) in ZooBank website, associated information of this work can be viewed. The LSID for this publication is: urn:lsid:zoobank.org:pub:2B03334F-27D3-4583-B057-2B5C1E321AEF.

## Supplementary Information

Supplementary Information.

## Data Availability

The data set generated from this study are available from the corresponding author upon reasonable request. Besides, Fig. 2c,d that support the findings of this study are available from Ms. Chutinun Mora but restrictions apply to the availability of these data, which were used under license for the current study, and so are not publicly available. Data are however available from the authors upon reasonable request and with permission of Ms. Chutinun Mora.

## References

[1] Baird W (1869). Remarks on several Genera of Annelides, belonging to the Group Eunicea, with a notice of such Species as are contained in the Collection of the British Museum, and a description of some others hitherto undescribed. Zool. J. Linn. Soc..

[2] Winterbourn M (1969). A freshwater nereid polychaete from New Zealand. NZ J. Mar. Freshw. Res..

[3] Uchida, H., Tanase, H. & Kubota, S. An extraordinarily large specimen of the polychaete worm *Eunice aphroditois* (Pallas) (Order Eunicea) from Shirahama, Wakayama, central Japan. (2009).

[4] Salazar-Vallejo SI, Carrera-Parra LF, de León-González JA (2011). Giant Eunicid Polychaetes (Annelida) in shallow tropical and temperate seas. Rev. Biol. Trop..

[5] Fauchald, K. A review of the genus *Eunice* (Polychaeta: Eunicidae) based upon type material. *Smithsonian Contributions to Zoology* (1992).

[6] Fauchald, K. & Jumars, P. A. The diet of worms: A study of polychaete feeding guilds. *Oceanogr. Mar. Biol. Ann. Rev.* **17**, 193–284 (1979).

[7] Lachat J, Haag-Wackernagel D (2016). Novel mobbing strategies of a fish population against a sessile annelid predator. Sci. Rep..

[8] Day, J.H. A monograph on the Polychaeta of Southern Africa. *British Museum of Natural History, Publication*, 1–878 (1967).

[9] Eriksson ME, Parry LA, Rudkin DM (2017). Earth’s oldest ‘Bobbit worm’—Gigantism in a Devonian eunicidan polychaete. Sci. Rep..

[10] Kielan-Jaworowska, Z. New Ordovician genera of polychaete jaw apparatuses. *Acta Palaeontol. Pol.* **7**(3–4), 291–332 (1962).

[11] Pemberton, S. G. & Frey, R. W. Trace fossil nomenclature and the *Planolites*-*Palaeophycus* dilemma. *J. Paleontol.* **56**(4), 843–881 (1982).

[12] Nara M (1995). *Rosselia socialis*: A dwelling structure of a probable terebellid polychaete. Lethaia.

[13] Belaústegui Z, de Gibert JM (2013). Bow-shaped, concentrically laminated polychaete burrows: A *Cylindrichnus* concentricus ichnofabric from the Miocene of Tarragona, NE Spain. Palaeogeogr. Palaeoclimatol. Palaeoecol..

[14] Rindsberg, A. K. & Kopaska-Merkel, D. C. *Treptichnus* and *Arenicolites* from the Steven C. Minkin paleozoic footprint site (Langsettian, Alabama, USA). Pennsylvanian Footprints in the Black Warrior Basin of Alabama. 1, 121–141 (2005).

[15] Gingras MK, Armitage IA, Pemberton SG, Clifton HE (2007). Pleistocene walrus herds in the Olympic Peninsula area: Trace-fossil evidence of predation by hydraulic jetting. Palaios.

[16] Pearson NJ, Gingras MK, Armitage IA, Pemberton SG (2007). Significance of Atlantic sturgeon feeding excavations, Mary's Point, Bay of Fundy, New Brunswick, Canada. Palaios.

[17] Gregory MR, Ballance PF, Gibson GW, Ayling AM (1979). On how some rays (Elasmobranchia) excavate feeding depressions by jetting water. J. Sediment. Res..

[18] Uchman A (2018). Feeding traces of recent ray fish and occurrences of the trace fossil *Piscichnus waitemata* from the Pliocene of Santa Maria Island, Azores (Northeast Atlantic). Palaios.

[19] Jensen S (1990). Predation by early Cambrian trilobites on infaunal worms-evidence from the Swedish Mickwitzia Sandstone. Lethaia.

[20] Kong D-Y, Lee M-H, Lee S-J (2015). Traces (ichnospecies *Oichnus paraboloides*) of predatory gastropods on bivalve shells from the Seogwipo Formation, Jejudo, Korea. J. Asia-Pac. Biodivers..

[21] Suppe J (1984). Kinematics of arc-continent collision, flipping of subduction and back-arc spreading near Taiwan. Geot. Soc. China Mem..

[22] Teng LS (1990). Geotectonic evolution of late Cenozoic arc-continent collision in Taiwan. Tectonophysics.

[23] Hong E, Wang Y (1988). Basin analysis of the Upper Miocene-Lower Pliocene series in northwestern foothills of Taiwan. Ti-Chih.

[24] Ho C, Hsu MY, Jen LS, Fong G (1964). Geology and coal resources of the northern coastal area of Taiwan. Bull. Geol. Surv. Taiwan.

[25] Teng LS, Hsiehz YW, Tanghz CH, Huanghz CY, Huang TC (1991). Tectonic aspects of the Paleogene depositional basin of northern Taiwan. Proce. Geol. Soc. China.

[26] Huang, C.S. Geological map of Taiwan scale 1:50,000-Taipei. *Geological map of Taiwan scale 1:50,000* (2005).

[27] Huang, C.S. & Liu, H.C. Geological map of Taiwan scale 1:50,000-Shuanghsi. *Geological map of Taiwan scale 1:50,000* (1988).

[28] Hong, E. The sedimentary environments of the Miocene-lower Pliocene series in northwestern foothills of Taiwan based on lithofacies and ichnofacies analyses. *PhD thesis*, 1–114 (National Taiwan University Department of Geology, 1988)

[29] Yu N, Teng L (1999). Depositional environments of the Taliao and Shihti Formations, northern Taiwan. Bull. Central Geol. Surv. Taiwan.

[30] Miguez-Salas, O., Löwemark, L., Pan, Y. Y. & Rodríguez-Tovar, F. J. Selective colonization after storm events in a delta environment: applied ichnology from the early Miocene of Taiwan. *Ichnos***(in Press)**.

[31] MacEachern, J. *et al.* The role of ichnology in refining shallow marine facies models. In *Recent advances in models of siliciclastic shallow-marine stratigraphy*, Vol. 90, 73–116 (Society for Sedimentary Geology (SEPM), Tulsa, 2008).

[32] Pemberton, S.G. *et al.* Shorefaces. In *Developments in sedimentology*, Vol. 64, 563–603 (Elsevier, Amsterdam, 2012).

[33] Frey RW, Pemberton SG (1985). Biogenic structures in outcrops and cores. I. Approaches to ichnology. Bull. Can. Petrol. Geol..

[34] Lalonde S, Dafoe L, Pemberton S, Gingras M, Konhauser K (2010). Investigating the geochemical impact of burrowing animals: Proton and cadmium adsorption onto the mucus lining of Terebellid polychaete worms. Chem. Geol..

[35] Zorn M, Gingras M, Pemberton S (2010). Variation in burrow-wall micromorphologies of select intertidal invertebrates along the Pacific Northwest coast, USA: Behavioral and diagenetic implications. Palaios.

[36] Jørgensen, B.B. & Nelson, D.C. Sulfi de oxidation in marine sediments: Geochemistry meets microbiology. In *Sulfur biogeochemistry: past and present*, Vol. 379, 63–81 (Geological Society of America, 2004).

[37] Schoonen, M.A. Mechanisms of sedimentary pyrite formation. *Special papers-Geological Society of America* 117–134 (2004).

[38] Frey RW, Howard JD, Pryor WA (1978). *Ophiomorpha*: Its morphologic, taxonomic, and environmental significance. Palaeogeogr. Palaeoclimatol. Palaeoecol..

[39] Hester N, Pryor W (1972). Blade-shaped crustacean burrows of Eocene age: A composite form of *Ophiomorpha*. Geol. Soc. Am. Bull..

[40] Löwemark L, Zheng Y-C, Das S, Yeh C-P, Chen T-T (2016). A peculiar reworking of *Ophiomorpha* shafts in the Miocene Nangang Formation, Taiwan. Geodin. Acta.

[41] Shimoda K, Tamaki A (2004). Burrow morphology of the ghost shrimp *Nihonotrypaea petalura* (Decapoda: Thalassinidea: Callianassidae) from western Kyushu, Japan. Mar. Biol..

[42] Bird F, Poore G (1999). Functional burrow morphology of *Biffarius arenosus* (Decapoda: Callianassidae) from southern Australia. Mar. Biol..

[43] Bromley RG (1996). Trace Fossils: Biology, Taxonomy and Applications.

[44] Winter, A.G., Deits, R.L. & Dorsch, D.S. Critical timescales for burrowing in undersea substrates via localized fluidization, demonstrated by RoboClam: a robot inspired by Atlantic razor clams. In *ASME 2013 International Design Engineering Technical Conferences and Computers and Information in Engineering Conference* DETC2013-12798, V06AT07A007 (American Society of Mechanical Engineers Digital Collection, 2013).

[45] Winter A, Deits R, Dorsch D, Slocum A, Hosoi A (2014). Razor clam to RoboClam: Burrowing drag reduction mechanisms and their robotic adaptation. Bioinspiration Biomimetics.

[46] Holland A, Dean J (1977). The biology of the stout razor clam *Tagelus plebeius*: I. Animal-sediment relationships, feeding mechanism, and community biology. Chesapeake Sci..

[47] Dashtgard, S.E. & Gingras, M.K. Marine invertebrate neoichnology. In *Developments in Sedimentology*, Vol. 64, 273–295 (Elsevier, Amsterdam, 2012).

[48] Winter AG, Deits RL, Hosoi AE (2012). Localized fluidization burrowing mechanics of *Ensis directus*. J. Exp. Biol..

[49] Gingras MK, Dashtgard SE, MacEachern JA, Pemberton SG (2008). Biology of shallow marine ichnology: A modern perspective. Aquat. Biol..

[50] Law CJ, Dorgan KM, Rouse GW (2014). Relating divergence in polychaete musculature to different burrowing behaviors: A study using Opheliidae (Annelida). J. Morphol..

[51] Kier WM (2012). The diversity of hydrostatic skeletons. J. Exp. Biol..

[52] Clark R (1981). Locomotion and the phylogeny of the Metazoa. Italian J. Zool..

[53] Verdonschot, P.F.M. Introduction to Annelida and the Class Polychaeta. In *Thorp and Covich's Freshwater Invertebrates* 509–528 (2015).

[54] Paxton H (2009). Phylogeny of Eunicida (Annelida) based on morphology of jaws. Zoosymposia.

[55] Hints O, Eriksson ME (2007). Diversification and biogeography of scolecodont-bearing polychaetes in the Ordovician. Palaeogeogr. Palaeoclimatol. Palaeoecol..

[56] Taylor A, Goldring R (1993). Description and analysis of bioturbation and ichnofabric. J. Geol. Soc..

[57] Nomenclature, I.C.o.Z. (2012). Amendment of Articles 8, 9, 10, 21 and 78 of the International Code of Zoological Nomenclature to expand and refine methods of publication. Bull. Zool. Nomencl..

