# Supplementary material for: The 20-million-year old lair of an ambush-predatory worm preserved in northeast Taiwan
Source: Sci Rep. 2021 Jan 21;11:1174. doi: 10.1038/s41598-020-79311-0 (PMC7820589; doi:10.1038/s41598-020-79311-0)
Supplement: Supplementary file 1 — Supplementary Information. [file 41598_2020_79311_MOESM1_ESM.pdf]

Supplementary Information

The 20-million-year old lair of an ambush-predatory worm preserved in northeast Taiwan

Yu-Yen Pan[1,7], Masakazu Nara[2], Ludvig Löwemark[1*], Olmo Miguez-Salas[3], Björn Gunnarson[4], Yoshiyuki Iizuka[5], Tzu-Tung Chen[6] & Shahin E. Dashtgard[7]

1. Department of Geosciences, National Taiwan University, P.O. Box 13-318, 106 Taipei, Taiwan
2. Department of Biological Sciences, Faculty of Science and Technology, Kochi University, Kochi 780-8520, Japan
3. Department of Stratigraphy and Palaeontology, University of Granada, 18002, Granada, Spain
4. Department of Physical Geography and Quaternary Geology, Stockholm University, SE-106 91 Stockholm, Sweden
5. Institute of Earth Sciences, Academia Sinica, Taipei, 11529, Taiwan
6. Department of Earth Sciences, University of Gothenburg, Box 460, SE 405 30 Göteborg, Sweden
7. Department of Earth Sciences, Simon Fraser University, Burnaby, British Columbia, Canada V5A 1S6

*Corresponding author: ludvig@ntu.edu.tw

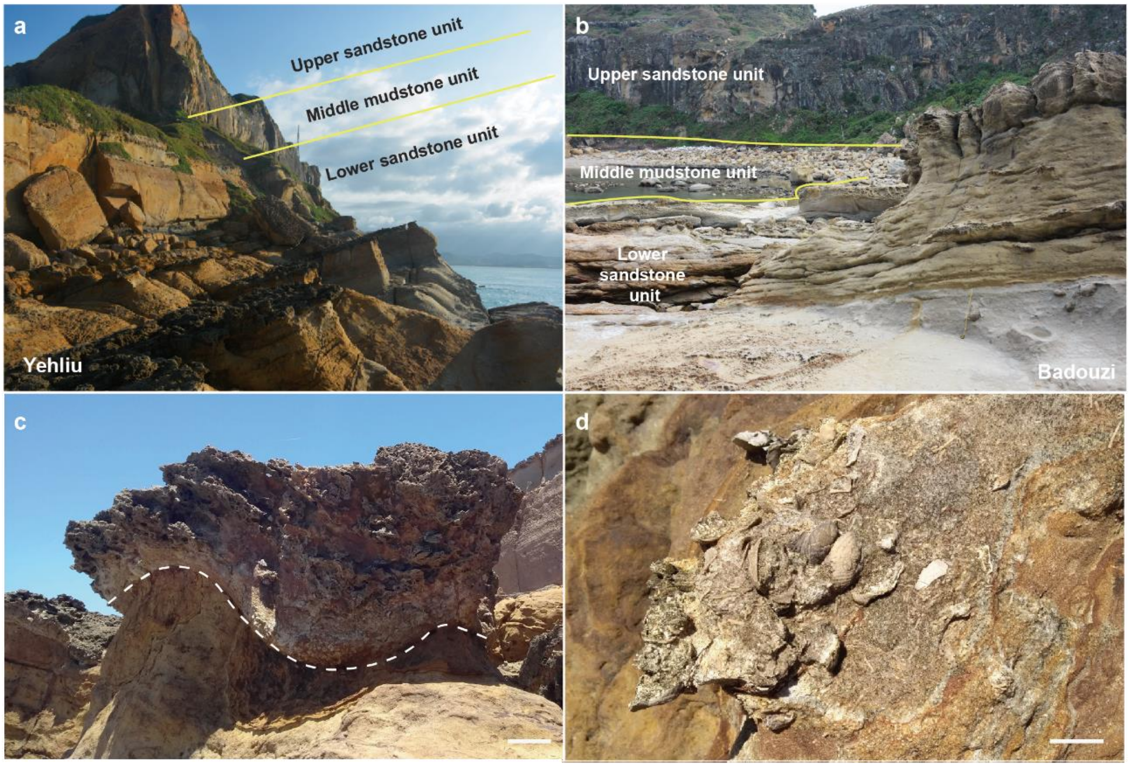

Supplementary Figure S1 Outcrops and shell debris-bearing beds in the study areas. Outcrops of the Yehliu Sandstone Member at **a** Yehliu Geopark, and **b** Badouzi promontory. The member is divided into an upper sandstone unit, middle mudstone unit and lower sandstone unit. *Pennichnus formosae* are concentrated in the lower sandstone unit where shell debris-bearing beds and bioturbated sandstones repeatedly occur. **c** Example of an erosion surface between bioturbated sandstone and shell debris-bearing bed from the Yehliu area. **d** Close up of broken shell debris and *Ditrupa* sp. within the shell debris-bearing bed shown in **c**. Scale bar: **c** = 20 cm; **d** = 2 cm.

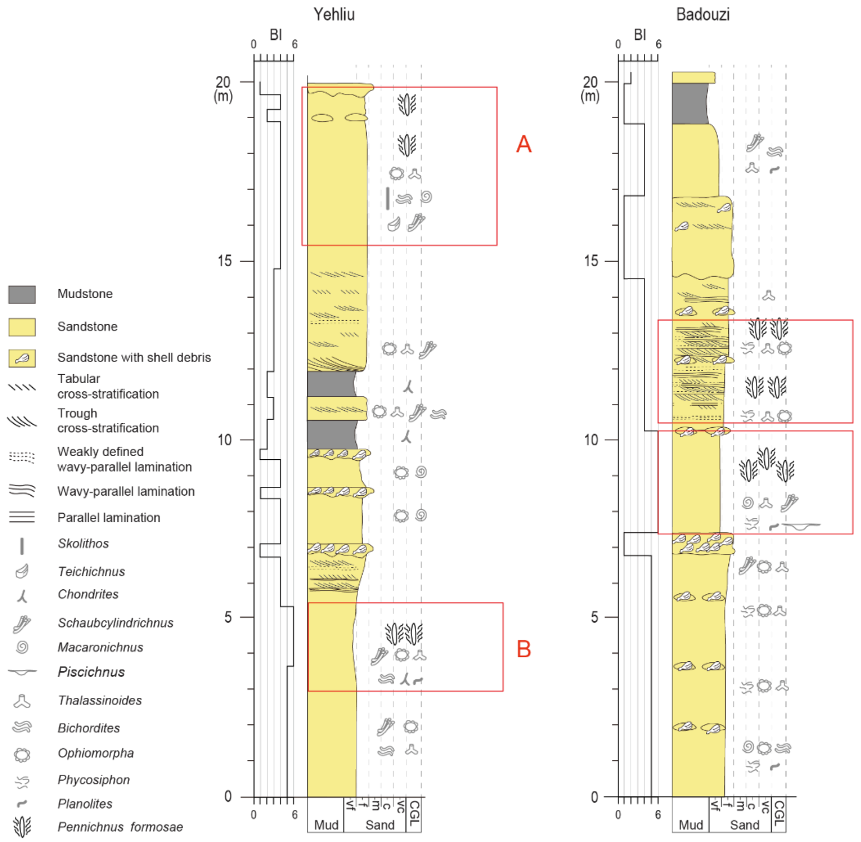

Zone	Trace fossil	Paleoenvironment	Density of *Pennichnus* (Number/ m^2)
A	**Dominant:** *Skolithos*, *Teichichnus*<br>**Sub-dominant:** *Ophiomorpha*	Proximal lower shoreface	0.5
B	**Dominant:** *Thalassinoides*, *Phycosiphon*, *Schaubcylindrichnus*, *Bichordites*<br>**Sub-dominant:** *Chondrites*, *Ophiomorpha*	Distal lower shoreface	0.7
C	**Dominant:** *Schaubcylindrichnus*, *Ophiomorpha*, *Phycosiphon*, *Bichordites*,<br>**Sub-dominant:** *Thalassinoides*, *Planolites*	Distal lower shoreface	0.8
D	**Dominant:** *Thalassinoides*, *Phycosiphon*, *Schaubcylindrichnus*, *Bichordites*, *Macaronichnus*, *Piscichnus*<br>**Sub-dominant:** *Ophiomorpha*, *Planolites*	Offshore	1.7

Supplementary Figure S2 Comparison between the study areas.

By comparing trace fossils, paleo-depositional environment, and density of *Pennichnus* between different sections within the stratigraphic columns, it turns out that *Pennichnus* specimens are more dominant in the offshore environment. The densities were quantified by dividing the number of specimens by outcrop area.

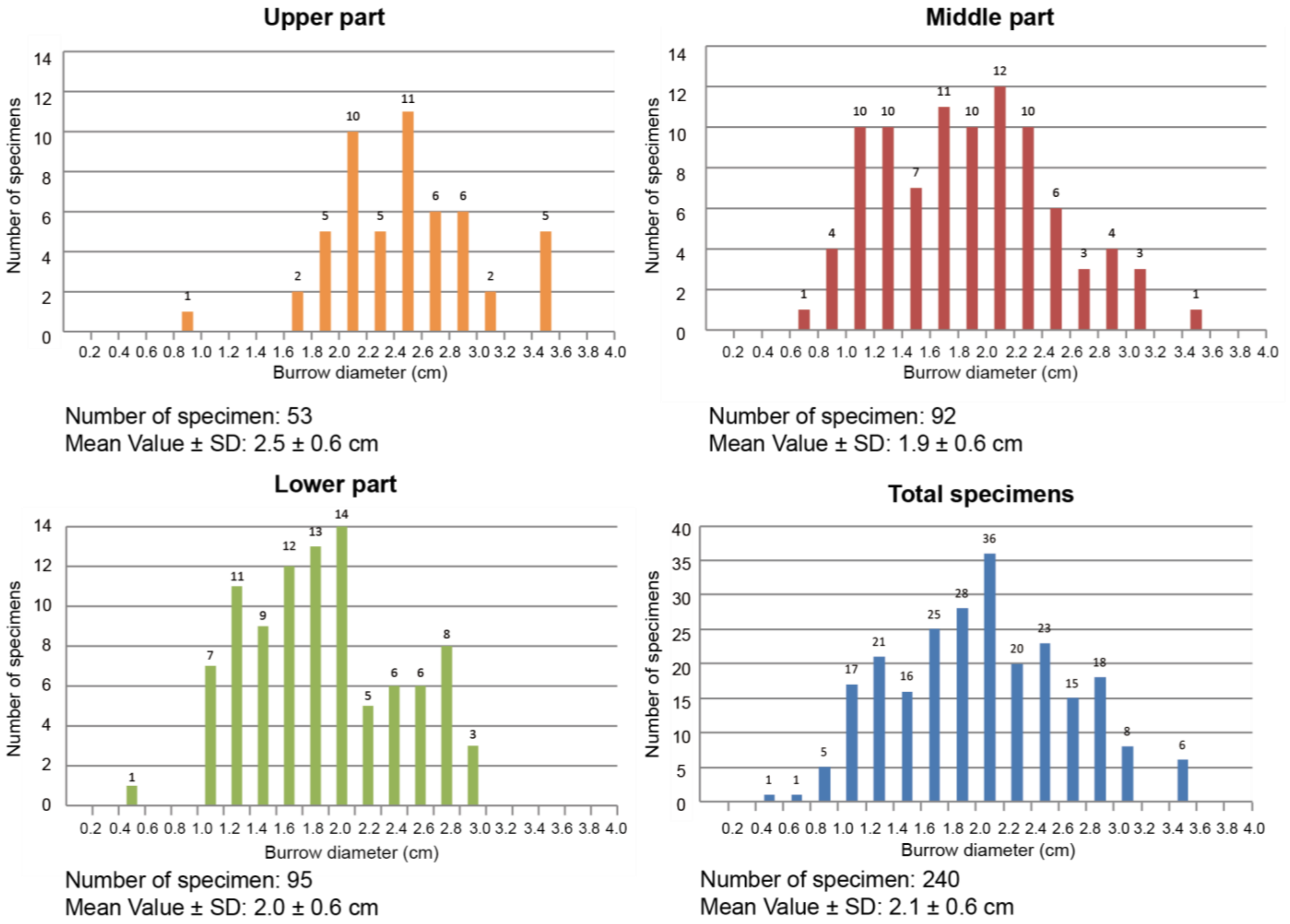

Supplementary Figure S3 Examination of the burrow diameter.

Comparison of the diameter of burrows from different parts of *Pennichnus* including the upper, middle, and lower part. In total, 240 specimens were measured.

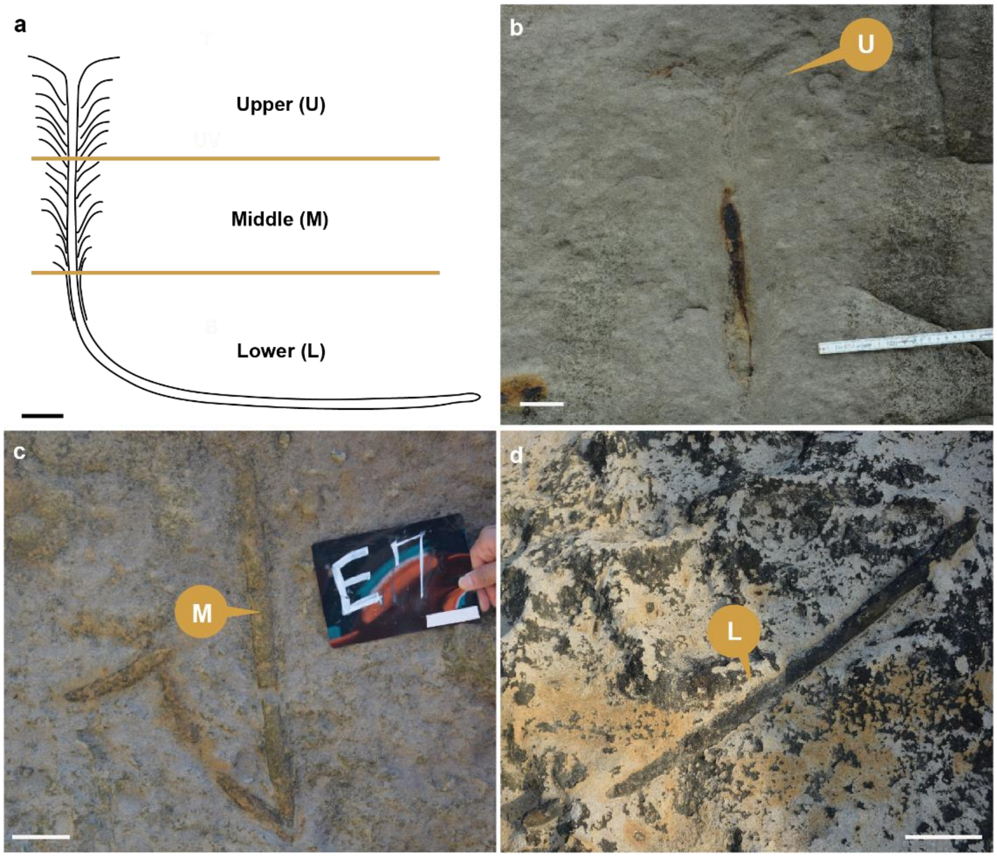

Supplementary Figure S4 Different portions of *Pennichnus*.

a Illustration of different exposed parts of *Pennichnus*. **b**, **c**, and **d** are the photos presenting the upper (U), the middle (M), and the lower part (L) of the specimen, respectively. Scale bar: **a** = 10 cm; **b–d** = 5 cm.

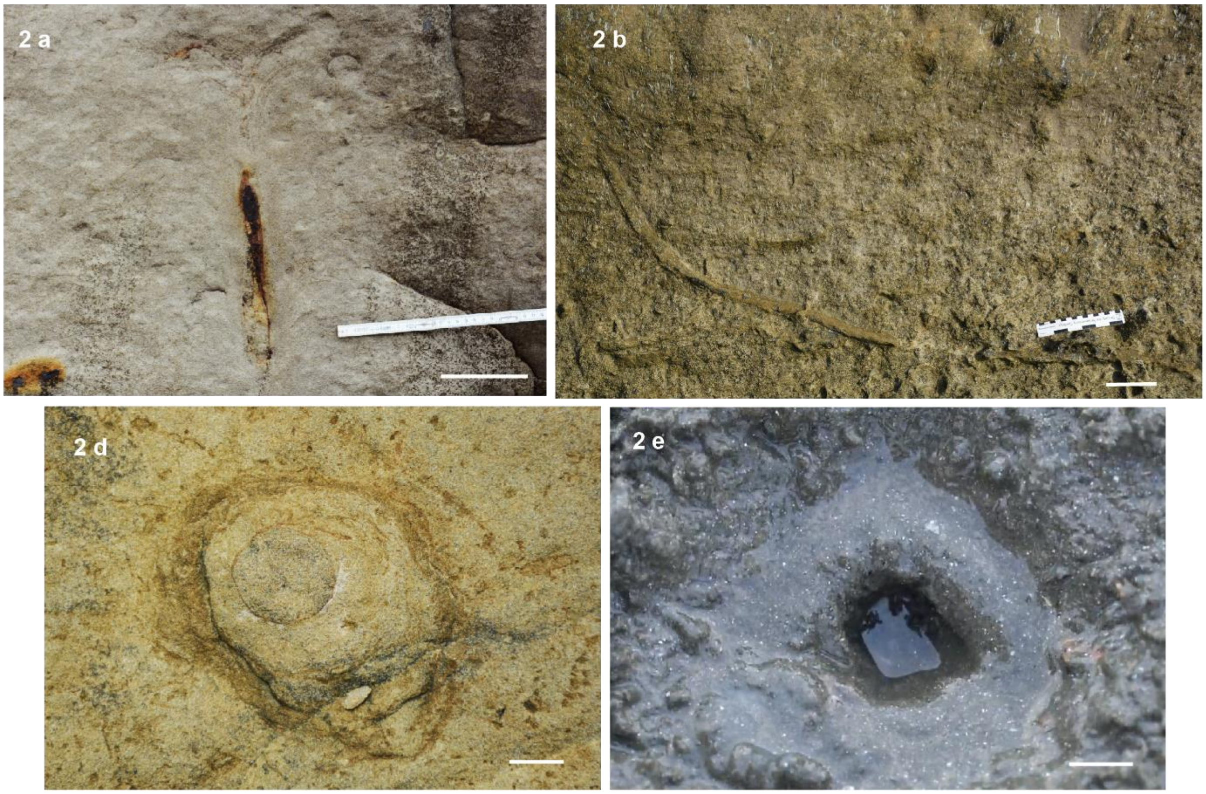

Supplementary Figure S5 Original photos of Figure 2.

Here are the original photos in Figure 2 and the tags on each photo correspond to their labels in Figure 2.

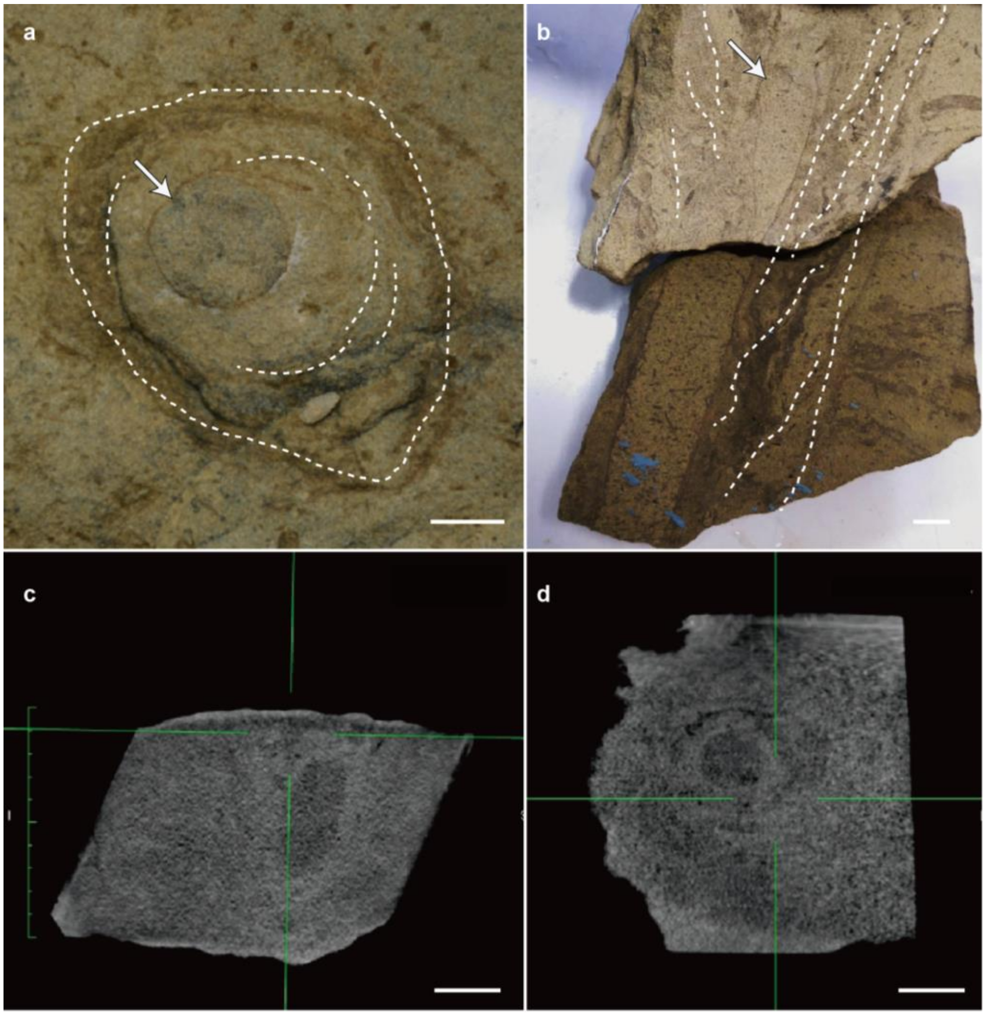

Supplementary Figure S6 Views of topmost *Pennichnus* from different angles.

a Plan view and **b** vertical section of the topmost part of *Pennichnus*. In both **a** and **b**, the white arrows mark the shaft while dashed white lines mark the disturbed sediment surrounding the burrow, suggesting that disturbed sediment around the burrow are preserved in a conical-shaped geometry. **c** CT scan of a funnel opening in the vertical section. **d** CT scan of concentric laminae around a funnel opening in the horizontal section. Scale bar: **a–b** = 2 cm; **c–d** = 3 cm.

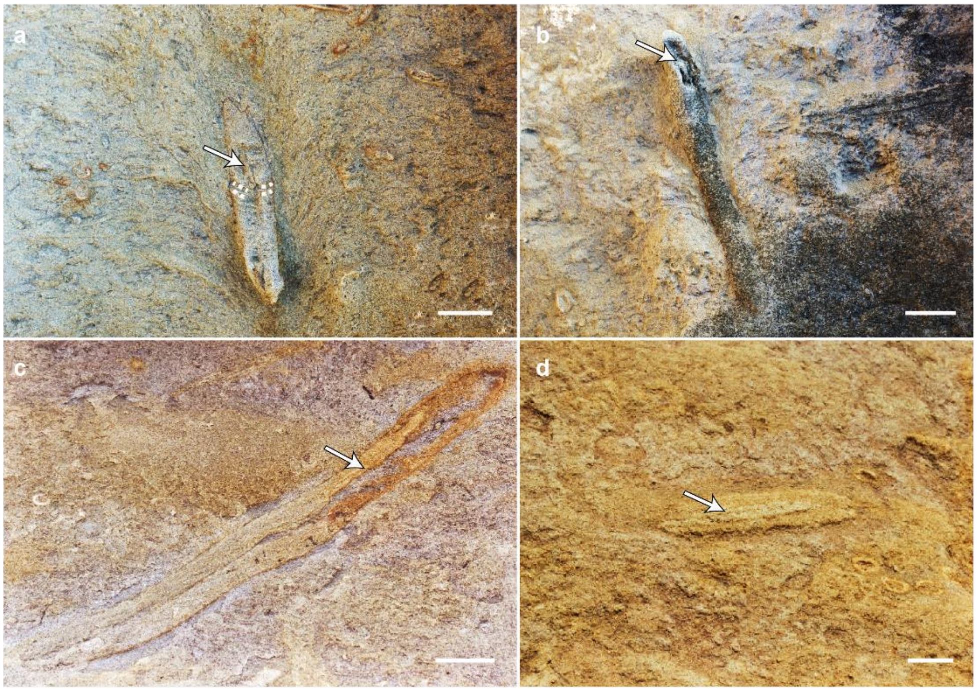

Supplementary Figure S7 *Pennichnus* specimens with aberrant features.

a Upper part of a *Pennichnus* specimen with an inner tube cutting through concave laminations (dashed white lines). **b** and **c** show the middle (bending) part of *Pennichnus* specimens containing an inner tube. **d** Lower (horizontal) part of a *Pennichnus* specimen with an inner tube. The white arrows point out the inner tube. Scale bar: **a–d** = 2 cm.

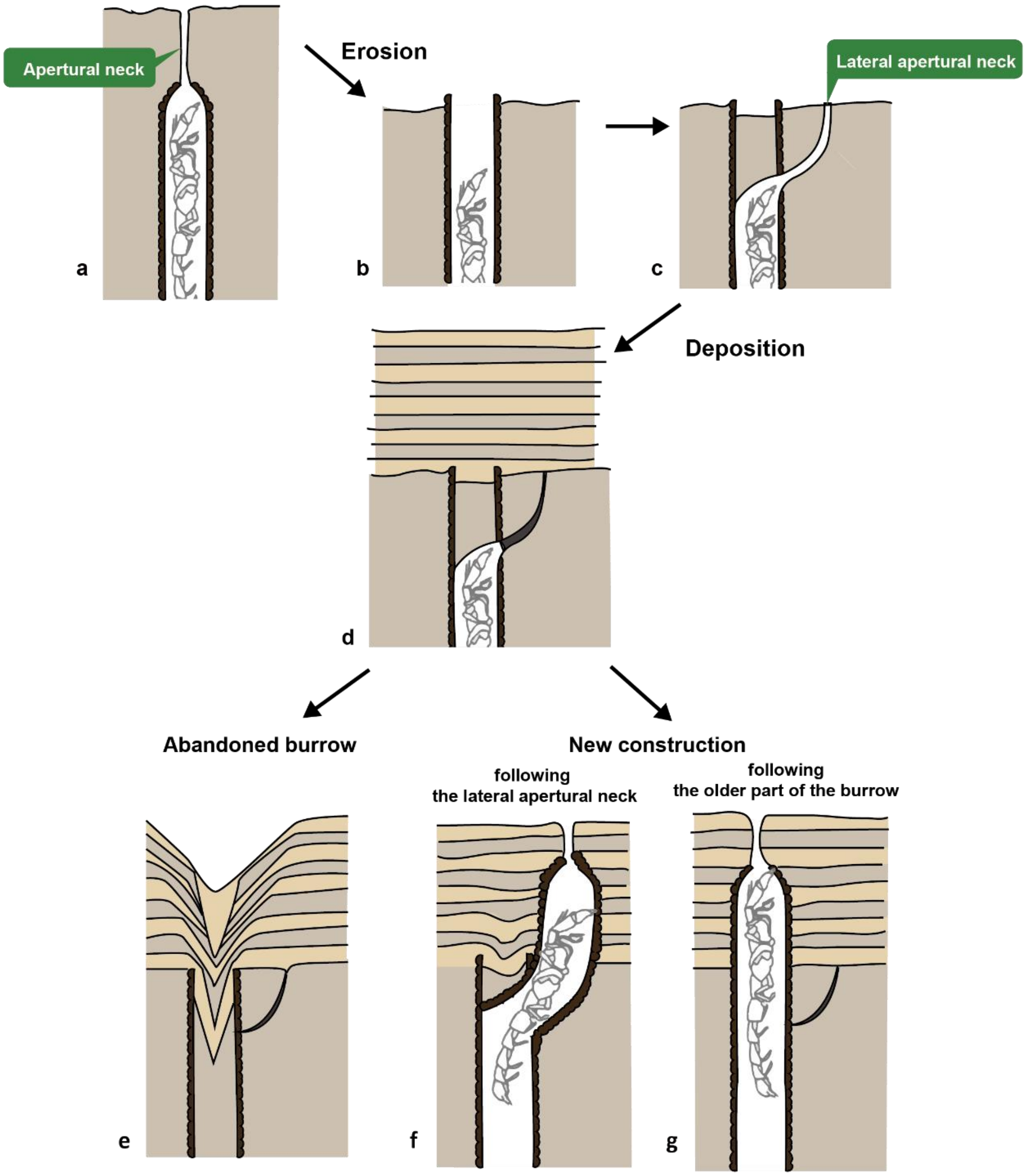

Supplementary Figure S8 Reponses and burrow morphology of *Callianassa major* to erosional damage and later burial of its burrow aperture

a A typical burrow of *Callianassa major* (producer of *Ophiomorpha*) with a constricted entrance (apertural neck). **b** The entrance is truncated by an erosional event. **c** The top is plugged by the animal and a new apertural neck is formed laterally. **d** Renewed sedimentation buries the damaged burrow. **e** The burrow is later abandoned by the trace maker leading to a collapse of entrance. **f** Producer keeps the burrow and extends upward following the lateral apertural neck. **g** Producer keeps the burrow and extends upward following the older part of the burrow. This illustration demonstrates that the funnel-shaped collapse of sediment only occurs above the burrow opening rather than along the entire upper part of the burrow as in *Pennichnus*. The illustration is modified from Frey et al., 1978[1].

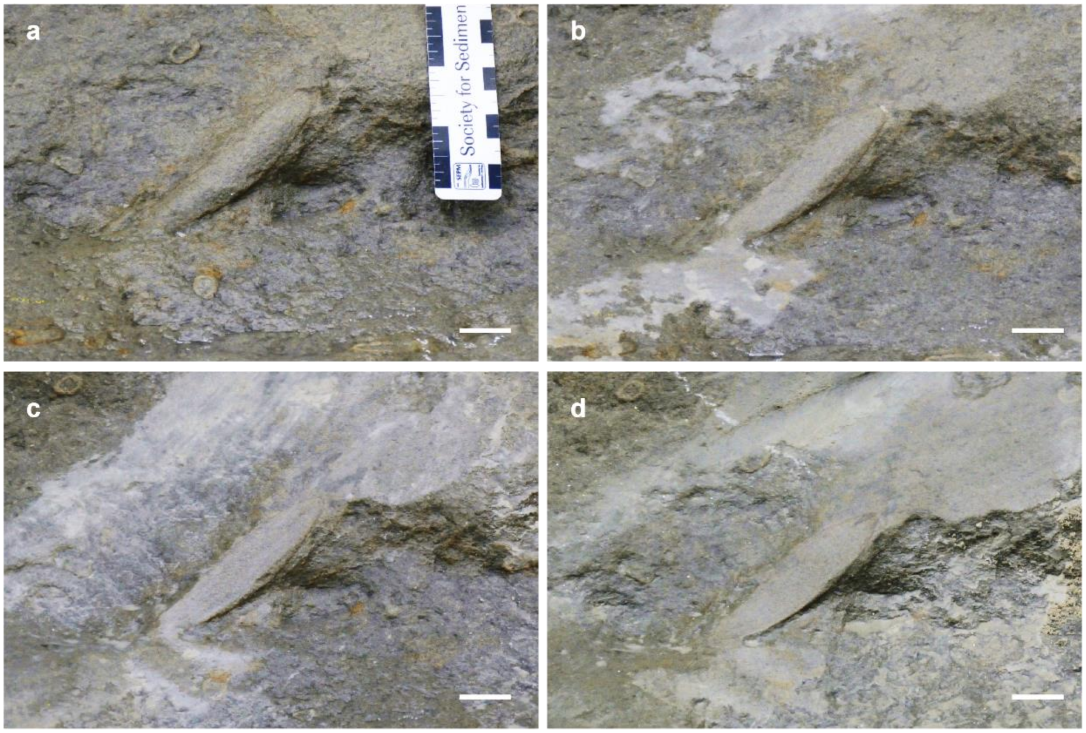

Supplementary Figure S9 Photos of *Pennichnus* specimen exposed by serial grinding.

From **a** to **d**, the specimen is ground down layer-by-layer with its internal gradually exposed. It is noteworthy that the serial grinding is an important approach to examine the internal structure in the field.

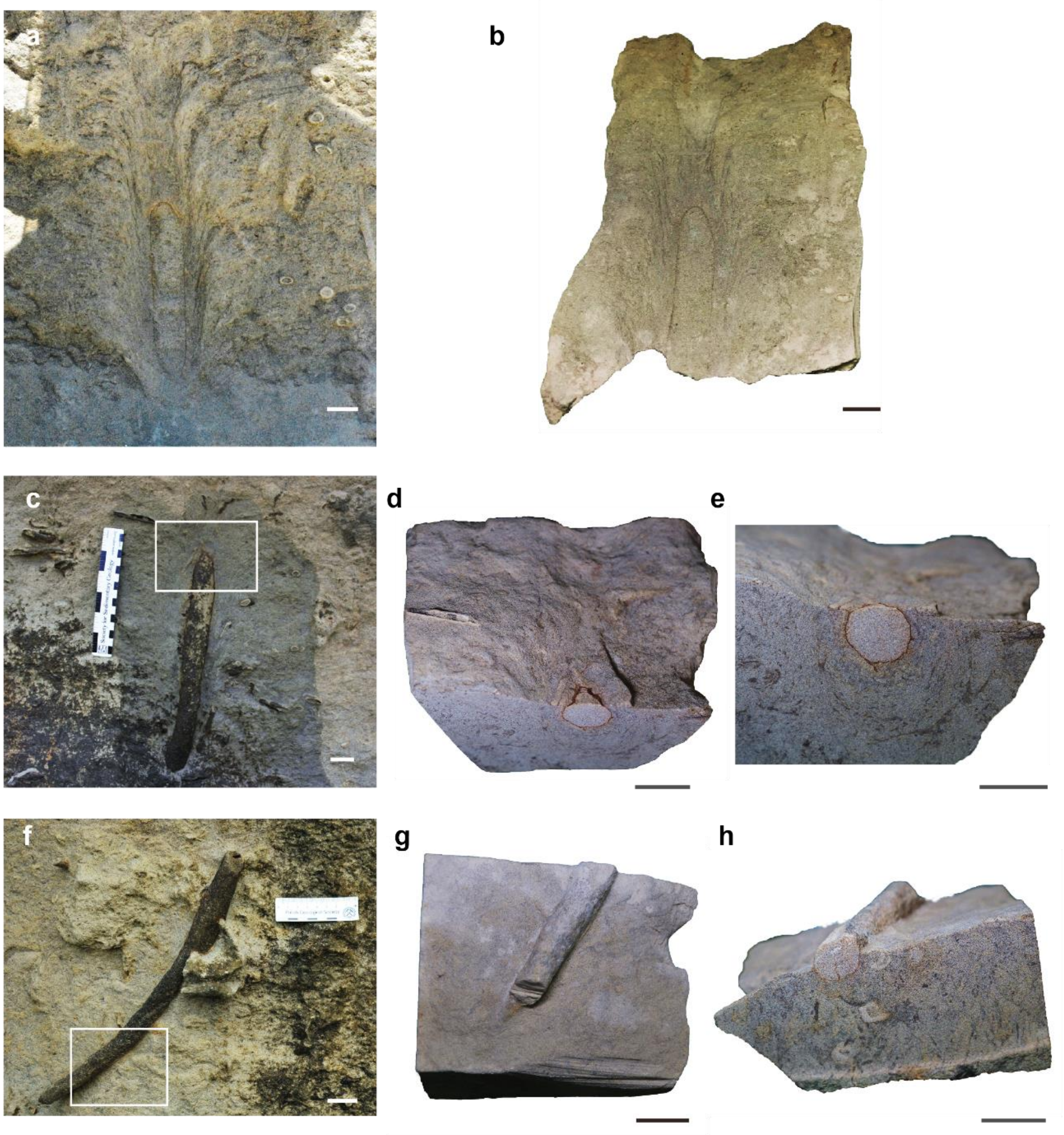

Supplementary Figure S10 Photos of *Pennichnus* type specimens.

a Holotype specimen (J01023) in the outcrop; **b** This sample constitutes the uppermost part of *Pennichnus* with obvious feather-like collapse structures around the burrow. **c** Paratype specimen (J01024) in the outcrop; **d** This sample belongs to the middle parts where feather-like structures are still visible and **e** concentric laminations surrounding the main burrow can be observed in the cross-section. **f** Paratype specimen (J01025) in the outcrop. This sample is composed of the curved, middle-to-lower part of the burrow; **g** Vertical view showing no feather-like structures around the burrow; **h** Horizontal cross-section of the burrow. White rectangles mark the sampled location. Scale bar: **a**–**h** = 3 cm.

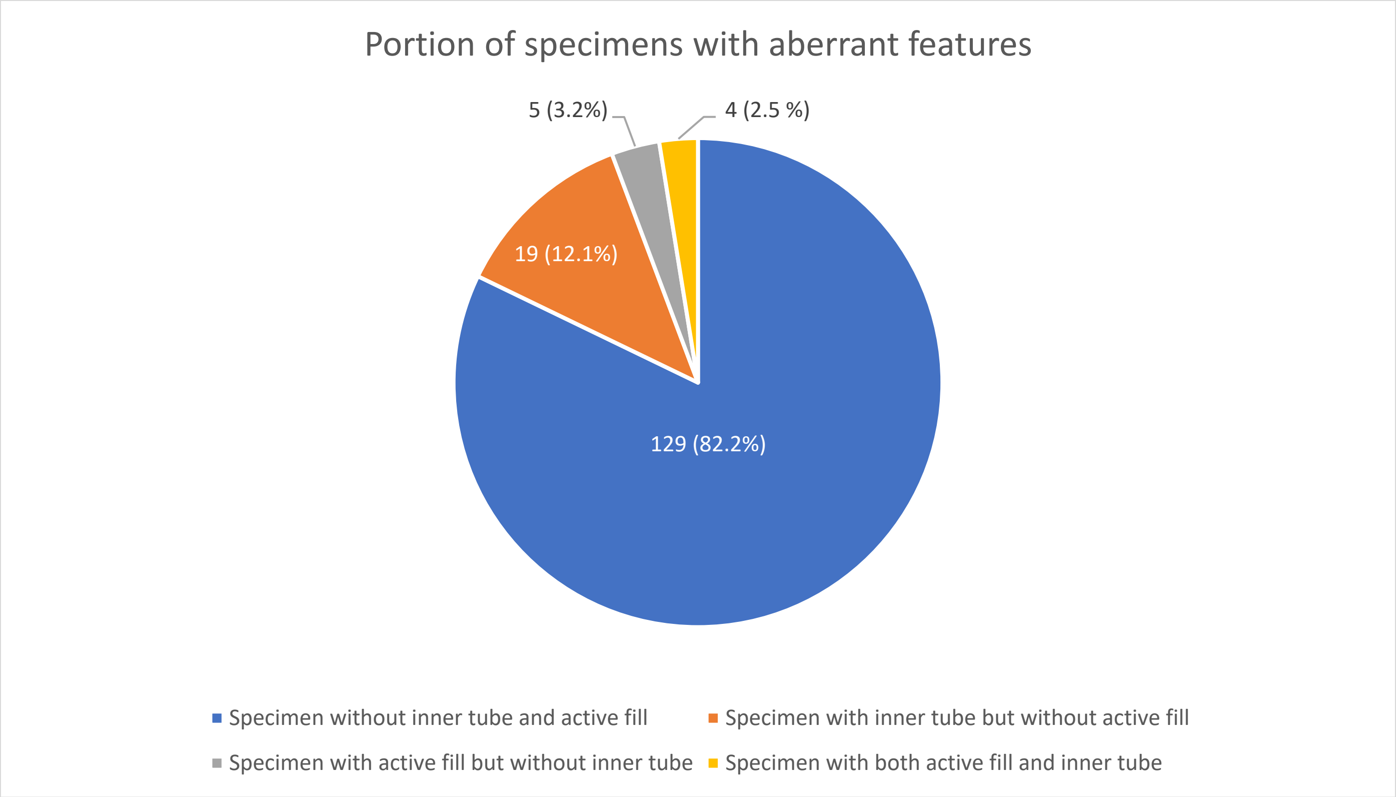

	Specimen without inner tube and active fill	Specimen with inner tube but without active fill	Specimen with active fill but without inner tube	Specimen with both active fill and inner tube
Yehliu	75	7	2	1
Badouzi	54	12	3	3
Sum	129	19	5	4

Supplementary Table 1 Portion of *Pennichnus* specimens with aberrant features.

Statistics of specimens with aberrant features. The calculation is based only on specimens whose internal structure were well-exposed (only 157 out of 319 specimens are recognized as well-exposed specimens).

Supplementary Discussion 1 Systematic ichnology

Ichnogenus PENNICHNUS new ichnogenus

Type and only ichnospecies. – *Pennichnus formosae* n. ichnospecies

LSID (Life Science Identifier) – urn:lsid:zoobank.org:pub:2B03334F-27D3-4583-B057-2B5C1E321AEF

Diagnosis. – L-shaped, thinly-lined, vertical-orientated, long burrow with a funnel top; circular to oval in horizontal cross section. Feather-like, inverted cone-in-cone structures surround the upper part of the shaft.

Etymology. – L. *Penna* = feather and L. *ichnus* =trace.

PENNICHNUS FORMOSAE new ichnospecies

Diagnosis. – Over 2-meter-long *Pennichnus* with 2–3 cm in diameter. The fill of burrow is essentially structureless.

Etymology. – L. *formosa*= beautiful or Republic of China (Taiwan); in reference to the occurrence place.

Holotype. – Specimen J01023 from the lower sandstone unit of the Yehliu Sandstone Member in Badouzi promontory (Supplementary Figs. S10a–b).

Paratype. – Specimens J01024 and J01025 from the lower sandstone unit of the Yehliu Sandstone Member in Badouzi promontory (about 10 m from the Holotype sampled location; Supplementary Figs. S10c-h).

Type localities– Taliao Formation, Miocene (Coordinate: 25°08'50.2"N, 121°47'51.5"E)

Repository. – The type specimens are deposited in the collections of the National Taiwan Museum, Taipei, Taiwan.

Description. – The burrow is vertical-oriented with even diameter (2–3 cm), slightly tapering downward. It usually penetrates straight through the strata over 70 cm and then gradually bends to horizontal, showing an overall L-shape. Total length of the trace fossil is approximately 2 m with maximum observed penetration 78 cm and maximum observed lateral extent 104 cm. The wall of the burrow is smooth and thinly lined with very fine grains. The fill of the burrow is structureless and disturbed by recolonization. The opening of the burrow displays a funnel shape. Around the upper part of the shaft, grayish feather-like, inverted cone-in-cone collapse structures occur, these structures gradually disappear downward along the shaft (Supplementary Fig. S6). In plan view, the aperture shows a circular to oval shape surrounded by concentric laminations.

Discussion. – There are several reasons to introduce *Pennichnus formosae* as a new ichnotaxon. While there are existing trace fossil consisting of cylindrical burrow with concentric laminae around, none of them contain all features observed in *Pennichnus*. For instance, *Rosselia*, the dwelling trace of a terebellid polychaete, shows a narrow cylindrical shaft surrounded by numerous concentric laminae[2]. However, in a complete *Rosselia* specimen, the laminations around the shaft is spindle-shaped rather than funnel-shaped in three-dimensions[2], conflicting with features of *Pennichnus*. Some crustacean trace fossils like *Ophiomorpha*[3], *Thalassinoides*[4], and *Psilonichnus*[5]consist of extensive, cylindrical burrows. However, *Ophiomorpha* and *Thalassinoides* are typically built in complicated, three-dimensional configuration with turn-around chambers[6]. Besides, although the configuration of U- and J-shaped *Psilonichnus* is somehow similar to *Pennichnus*, lateral branches are commonly shown in *Psilonichnus*[5]. Sometimes, V-in-V collapsing laminae would form when sediment entered eroded, abandoned shrimp burrow, however, these laminae occur above the burrow opening but don't continue downward along the burrow[1]. This is because these collapsing laminae simply result from sediment collapsing into truncated burrow rather than the producer's reciprocating movement which contributes to continual collapse and re-establishment of the burrow. *Dolopichnus*[7], the dwelling trace of sea anemone, consists of a thinly lined cylindrical tube with structureless fill surrounded by downward laminae, which shows a similarity to the upper part of *Pennichnus*. However, sea anemones usually produce a vertical burrow rather than L-shaped burrow. While nested laminae occur in *Conichnus*, the escape traces produced by sea anemones or bivalves[8,9], are similar to feather-like structures, these downward deflected laminae usually extend across, rather than surround the burrow[10]. Since escape traces are made in response to fast sediment aggradation, their burrow walls are usually blurry or unlined. Hence, feather-like structures preserved in *Pennichnus* can't be satisfactorily explained by escape movements. *Lingulichnus hamatus*[11] , the trace fossil produced by Lingulid brachiopods during vertical re-adjustment of their burrows[11,12], is composed of a vertically orientated, unbranched, curving burrow. Nevertheless, since this trace fossil is built by temporary re-establishment, the burrow is unlined. Although some fast-burrowing bivalves can produce slender burrows with a funnel top, their almond- or keyhole-shaped burrows in plan view and the blurry burrow wall constitute a significant difference from *Pennichnus*. Vermiform vertebrates such as garden eels are known to produce a long burrow with a funnel-shaped aperture in loose sediment. However, unlike the straight *Pennichnus* burrow segments, garden eels' burrows show a vertical sinuous curve-shape attributed to the anguilliform motion[13], which is the typical axial-based undulatory locomotion of elongate aquatic vertebrates[14]. As a result, *Pennichnus* is most likely produced by a giant vermiform invertebrate like a giant polychaete. Total lack of smaller-sized *Pennichnus* strongly suggests that the trace maker changed its behavior through ontogeny, and the Bobbit worm (*Eunice aphroditois*) is known to be free-living in its juvenile stages and burrow when reaching mature stage[15], further supporting the presented hypothesis.

Ethology. –The trace fossil represents praedichnial ethology and were inhabited by giant vermiform invertebrates. The funnel-like top and the distinct feather-like structures reveal the reciprocating movement of the producer. The most likely trace maker of *Pennichnus formosae* are ambush predatory worms (e.g. *Eunice aphroditois*), who support the long burrow with mucous, as their slender bodies repeatedly thrusted out hunting for prey. The struggle of the prey and the retreat of the worm further disturb surrounding sediment, enhancing the conical feather-like structures.

Environment. –Mainly distributed in distal lower shoreface to offshore environments.

Supplementary Reference:

1. Frey, R.W., Howard, J.D. & Pryor, W.A. *Ophiomorpha*: its morphologic, taxonomic, and environmental significance. *Palaeogeography, Palaeoclimatology, Palaeoecology* **23**, 199-229 (1978).
2. Nara, M. *Rosselia socialis*: a dwelling structure of a probable terebellid polychaete. *Lethaia* **28**, 171-178 (1995).
3. Lundgren, B. Stndier öfvor fossilförande lösa block. *Geologiska Föreningen i Stockholm Förhandlingar* **13**, 111-121 (1891).
4. Ehrenberg, K. Ergänzende Bemerkungen zu den seinerzeit aus dem Miozän von Burgschleinitz beschriebenen Gangkernen und Bauten dekapoder Krebse. *Paläontologische Zeitschrift* **23**, 354-359 (1944).
5. Frey, R.W., Curran, H.A. & Pemberton, S.G. Tracemaking activities of crabs and their environmental significance: the ichnogenus *Psilonichnus*. *Journal of Paleontology*, 333-350 (1984).
6. Bird, F. & Poore, G. Functional burrow morphology of *Biffarius arenosus* (Decapoda: Callianassidae) from southern Australia. *Marine Biology* **134**, 77-87 (1999).
7. Alpert, S.P. & Moore, J.N. Lower Cambrian trace fossil evidence for predation on trilobites. *Lethaia* **8**, 223-230 (1975).
8. Desai, B.G. & Saklani, R.D. Palaeocommunity dynamics and behavioral analysis of *Conichnus*: Bhuj Formation (Lower Cretaceous), Kachchh-India. *Ichnos* **22**, 43-55 (2015).
9. Gingras, M.K., Dashtgard, S.E., MacEachern, J.A. & Pemberton, S.G. Biology of shallow marine ichnology: a modern perspective. *Aquatic Biology* **2**, 255-268 (2008).
10. Savrda, C. Equilibrium responses reflected in a large *Conichnus* (Upper Cretaceous Eutaw formation, Alabama, USA). *Ichnos* **9**, 33-40 (2003).
11. Zonneveld, J.-P. & Pemberton, S.G. Ichnotaxonomy and behavioral implications of lingulide-derived trace fossils from the Lower and Middle Triassic of Western Canada. *Ichnos* **10**, 25-39 (2003).
12. Savazzi, E. Burrowing in the inarticulate brachiopod *Lingula anatina*. *Palaeogeography, Palaeoclimatology, Palaeoecology* **85**, 101-106 (1991).
13. Tyler, J.C. & Smith, C.L. Systematic significance of the burrow form of seven species of garden eels (Congridae, Heterocongrinae). American Museum novitates; no. 3037. (1992).
14. Gillis, G.B. Undulatory locomotion in elongate aquatic vertebrates: anguilliform swimming since Sir James Gray. *American Zoologist* **36**, 656-665 (1996).
15. Day, J.H. A monograph on the Polychaeta of Southern Africa. *British Museum of Natural History, Publication*, 1-878 (1967).
